# A Systematic Review of the Prevalence of Gestational Diabetes in Norway

**DOI:** 10.3390/ijerph18041423

**Published:** 2021-02-03

**Authors:** Samira Behboudi-Gandevani, Ranjan Parajuli, Mojtaba Vaismoradi

**Affiliations:** Faculty of Nursing and Health Sciences, Nord University, 8049 Bodø, Norway; ranjan.parajuli@nord.no (R.P.); mojtaba.vaismoradi@nord.no (M.V.)

**Keywords:** gestational diabetes mellitus (GDM), health care, Norway, public health, prevalence, reproductive health, systemic review

## Abstract

Gestational diabetes mellitus (GDM) is a major public health problem and a threat to maternal and child health. There is a lack of integrated and systematically synthesized knowledge about the prevalence GDM in Norway. Therefore, this systemic review aimed to present the best available peer-reviewed published evidence from the past 20 years about the prevalence of GDM in Norway. A comprehensive literature search was performed on online databases consisting of PubMed (including Medline), Web of Science, and Scopus to retrieve original research articles published on the prevalence of GDM up to August 2020. Also, databases of Norart and SveMed^+^ in the Norwegian language were searched to enhance the search coverage. Data were extracted using a standardized protocol and data collection form and were presented narratively. A total of 11 studies were selected to include for data analysis and synthesis with the total sample size of 2,314,191 pregnant women. The studies included heterogenous populations and mostly reported the prevalence of GDM in healthy mothers with singleton pregnancies. The prevalence of GDM in population registered-based studies in Norway was reported to be lower than 2%, using the World Health Organization (WHO) 1999 criteria. However, studies on high-risk populations such as the non-European ethnicity reported prevalence rates ranging from 8% to 15%. Given the evidence from available literature that reported trends in the prevalence of GDM, an increase in the prevalence of GDM across most racial/ethnic groups studied in Norway was observed. Overall, the prevalence of GDM in the low risk population of Norway is fairly low, but the available literature supports the perspective that the prevalence of GDM has shown an increasing trend in recent decades. This finding is very important for health service planning and evaluation, policy development, and research in Norway. Large-scale prospective studies, using the national data, are warranted to provide firm evidence over coming years. Our review findings can help policy makers devise appropriate strategies for improving women’s reproductive health.

## 1. Introduction

Gestational diabetes mellitus (GDM) is considered a major pregnancy complication [1]. It has been defined as glucose intolerance in the second or third trimester of pregnancy in women who have had not clearly overt diabetes prior to gestation [2]. This is one of the most common endocrinopathies affecting 4–12% of all pregnancies. However, its prevalence is raised depending on diagnostic criteria and the presence of different risk factors such as maternal age and body mass index (BMI); prevalence of overt diabetes; population ethnicity; genetic, social, and environmental factors [3,4,5,6].

The etiology of GDM is multifactorial and has not been completely understood. In most patients with GDM, gestational hyperglycemia is the result of impaired glucose tolerance due to pancreatic β-cell dysfunction and post-receptor defects in the insulin signaling cascade, as the background of chronic insulin resistance [7,8]. It is followed by progressive insulin resistance due to the increased placental production of diabetogenic hormones including estrogen, progesterone, and human placental lactogen during the second trimester of pregnancy [9,10]. However, GDM carries a serious risk of feto-maternal, neonatal mortality and morbidity [11], and the lifelong risk of obesity, type 2 diabetes mellitus and cardiovascular diseases in the mother and child later in life [12,13,14,15].

During the last 20 years, the prevalence of GDM has increased worldwide and it is expected to continue to rise along with an increase in advanced maternal age, pre-conception obesity and impaired glucose tolerance among women [16,17]. Moreover, the use of the universal screening strategy and the more stringent diagnostic criteria for GDM lead to a higher prevalence of GDM among pregnant women and potentially increase the costs of health care [18].

Nordic countries as well have shown a similar trend in increasing the aforementioned risk factors [19,20]. Specifically in Norway, the mean of maternal age at first birth in the recent two decades has been more than 25 years with an increasing trend from 27.3 years in 2000 to 29.8 years in 2019 with the most pronounced in the recent decade [21], which can influence the prevalence of GDM and other related health outcomes. In addition, it has been reported that 4.7% of the Norwegian population have a diabetes diagnosis. Although this is fairly low compared to the global scale, it has shown an increasing trend as well [22].

According to the Norwegian guidelines, the target-based screening approach has been recommended for high-risk pregnant women including those with a maternal age older than 25 years, previous history of GDM, macrosomia greater than 4500 g, shoulder dystocia, first relative’s overt diabetes history, Asian and African ethnicity and those with a BMI more than 30 kg/m^2^ [23]. In this respect, pregnant women are set to have two screening phases as follows: (i) measurement of Hb A1C at the first trimester of pregnancy before the 16 weeks of gestation for high risk pregnant women to find any kind of undiagnosed hyperglycemia, and (ii) oral glucose tolerance test (OGTT) with 75 g glucose at the 24–28 weeks of gestation for high-risk pregnant women who have the normal glycemia condition at the first-trimester screening.

For the last two decades in Norway, GDM has been defined based on the WHO-1999 criteria as follows: fasting blood glucose level (FBS) ≥ 7.0 mmol/L and/or OGTT 75 g 2 h level of blood glucose (BS-2h) ≥ 7.8 mmol/L [23]. In 2017, the Norwegian institute of public health revised the GDM definition and suggested the lower diagnostic glucose threshold compared to the previously used criteria [24]. According to the new guideline, GDM has been defined as the FBS level between 5.3 and 6.9 mmol/l and/or a BS-2h level between 9.0 and 11.0 mmol/l after a 75 g OGTT. The latest national report in 2019 estimated that the prevalence of GDM in Norway ranges between 3% and 8% using the WHO 1999 criteria and 10% or maybe even more based on the new criteria [24]. In addition, some studies indicate concerns about the underestimation of the prevalence of GDM in the general population [25,26]. Therefore, the aim of this systemic review was to present the best available peer-reviewed published evidence about the prevalence of GDM in Norway in order to provide an up-to-date overview of the current knowledge about GDM.

## 2. Materials and Methods

### 2.1. Design

A systematic review of national and international literature was conducted that was informed by the Preferred Reporting Items for Systematic Reviews and Meta-Analyses (PRISMA) [27].

### 2.2. Eligibility Criteria

Appropriate studies for inclusion to data analysis and knowledge synthesis were original research studies, focused on the prevalence of GDM in Norway, published in scientific peer-reviewed journals and in English and Norwegian language. Therefore, non-original studies including reviews, commentaries, editorials, letters, conference proceedings, case reports that did not provide accurate and clear data were excluded. In addition, studies that reported on similar study populations at a similar point in time were excluded.

### 2.3. Search Strategy

A comprehensive literature search was conducted on online databases such as PubMed (including Medline), Web of Science and Scopus to retrieve all relevant articles published up to August 2020. Also, the databases of Norart and SveMed^+^ in Norwegian language were searched to enhance the search coverage. Further, a manual search in the references list of selected studies and other relevant reviews was performed to maximize the identification of eligible studies.

The following keywords, alone or in combination, using the Boolean method, were used to carry out the search: (gestational diabetes OR gestational diabetes mellitus OR GDM OR pregnancy induced diabetes OR gestational hyperglycemia OR gestational glucose intolerance) AND (incidence OR prevalence OR epidemiology) AND (Norway OR Norwegian OR Scandinavia OR Scandinavian). Support with the search process was achieved from an expert librarian.

### 2.4. Study Selection and Data Extraction

The screening of titles, abstracts and full text of retrieved articles based on the eligibility criteria was performed independently by the authors. For each eligible study, a predetermined data extraction form was used to collect the following data: author’s name, journal, publication year, study design, data resources, sample size, population characteristics including age and BMI, GDM screening strategy, GDM criteria and laboratory values of blood sugar tests, study quality assessment and outcome measurement including the number and prevalence of GDM. Discussions were held by the authors to check the search process and resolve disagreements on the inclusion of selected studies. Given heterogeneities in the methods, objectives, and results of the selected studies, they did not lend themselves to meta-analysis. Therefore, the review results were described narratively.

### 2.5. Quality Assessment and Risk of Bias

The quality of the studies was critically appraised in terms of methodological structure and presentation of results. Two members of the research team who were blinded to study author, journal name and institution evaluated the quality of the studies independently. The quality of observational studies was assessed using the modification of the Newcastle–Ottawa Quality Assessment Scale for Non-Randomized Studies (NRS) [28] regarding selection, comparability and outcomes. Studies with scores above 6 were considered high quality, 4–6 moderate quality, and less than 4 low quality. The risk of bias in observational studies was assessed using the Risk of Bias in Non-Randomized Studies (ROBINS) tool. Five domains related to risk of bias were assessed in each cross-sectional study including: bias in the assessment of exposure, development of the outcome of interest in case and controls, bias in the selection of cases, selection of controls, and control of prognostic variables. In addition, seven domains related to risk of bias were assessed including bias in the selection of exposed and non-exposed cohort, assessment of exposure, presence of the outcome of interest at the start of the study, the control of prognostic variables, assessment of the presence or absence of prognostic factors, assessment of outcome, and adequacy regarding the follow up of cohorts. Authors’ judgments were categorized as ‘low risk’, ‘high risk’, and ‘unclear risk’.

## 3. Results

### 3.1. Search Results and Study Selections

**Figure 1:**
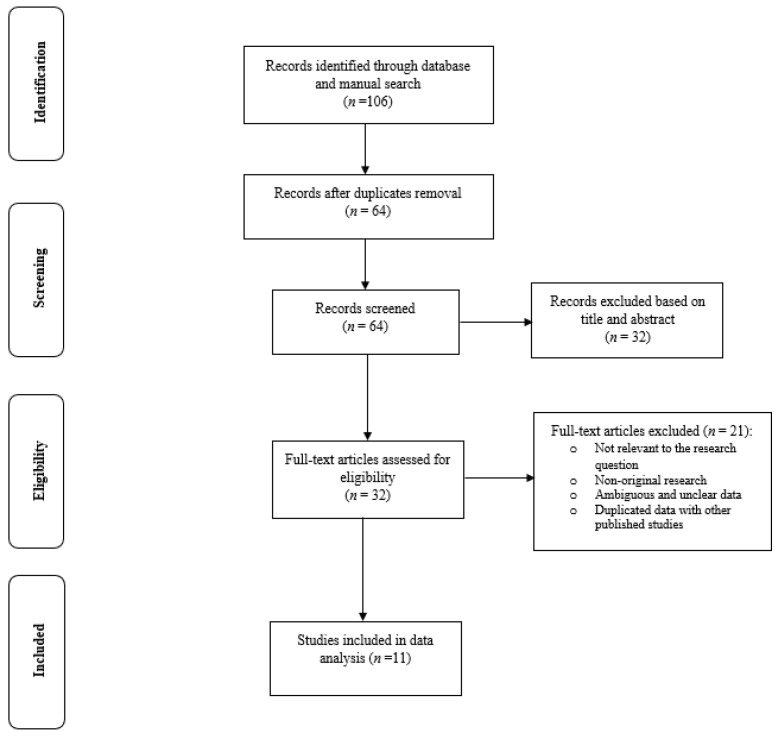
Flow diagram of the study process.

The search strategy yielded 106 potentially relevant articles, of which 32 articles were identified for further full-text assessment. Finally, 11 studies [26,29,30,31,32,33,34,35,36,37,38] consisting of data from 2,314,191 pregnant women were selected for inclusion to the data analysis (Figure 1). The full-text appraisal of the studies did not lead to the exclusion of any study as they all had the acceptable quality in terms of the research structure and relevance to the review aim. Therefore, all selected studies were included to the data analysis and knowledge synthesis.

### 3.2. General Characteristics of the Selected Studies

The characteristics of the selected studies assessing the prevalence of GDM in Norway were presented in Table 1. Among the studies included, 9 (81.8%) used a population-based design [26,30,32,33,34,35,36,37,38]. A total of 6 (54.5%) studies used the national data of Medical Birth Registry of Norway [30,32,33,34,37,38] and other studies used data from local hospitals and public health clinics in Oslo, the capital city [26,29,35], Trondheim and Stavanger [31], and Nordland and Tromsø [36]. All studies used the national criteria for the diagnosis of GDM. In addition to national definition, two (18.1%) studies used the International Association of Diabetes and Pregnancy Study Group (IADPSG) or Modified IADPSG criteria [26,31], one (9%) the WHO-2013 criteria [35] and one (9%) the 2017 revised national criteria [35] for the diagnosis of GDM. The studies’ population was heterogenous, but they mostly reported the prevalence of GDM in healthy mothers with singleton pregnancies lasting more than 22 weeks of gestation.

### 3.3. Prevalence of Gestational Diabetes Mellitus (GDM) in Norway

Some regional and registered studies were conducted in the different parts of Norway. The reported range of GDM, according to the selected studies, varied widely with a range as low as 0.7% [37] to as high as 31.5% [26], mostly due to various GDM diagnostic criteria, ethnicity of participants, data sources and the population-based design of studies. In this respect, using the national criteria, the reported prevalence of GDM varied widely, ranging from 0.9% to 13.4% [26,29,31,32,33,34,36,38]. However, the use of the strictest criteria of IADPGS and WHO 2013 sharply increased the prevalence of GDM from 7.4% to 31.5% [26,31,35]. On the other hand, use of the 2017-revised national criteria, it was estimated that 9.2% of pregnant women had GDM [35].

Among these population-based studies conducted in Norway, the prevalence of GDM was approximately 13% in one study [26], and 1–2% in other studies [30,32,33,34,36]. The prevalence of GDM in population registered-based studies in Norway is lower than 2%, using the World Health Organization (WHO) 1999 criteria [32,33,34,37,38]. While studies in high-risk populations, such as the non-European ethnicity, reported prevalence rates ranging from 8% to 15% [26,36,38]. Jenum, et al. (2012) in a population-based study reported that GDM was more prevalent in non-Western Europeans minority groups from south, east and middle east Asia, eastern Europe, Somalia, sub-Saharan Africa and south America than the Western European pregnant women, (15% vs. 11%) using the national criteria and (37% vs. 24%) using the modified IADPSG definition for GDM, respectively [26]. Leeves, et al. (2019) in a hospital-based study of two northern cities of Norway indicated that pregnant women of non-European ethnicity had a greater risk of developing GDM compared to the European ethnicity pregnant women as 14.7% vs. 2.2%, respectively [36].

**Figure 2:**
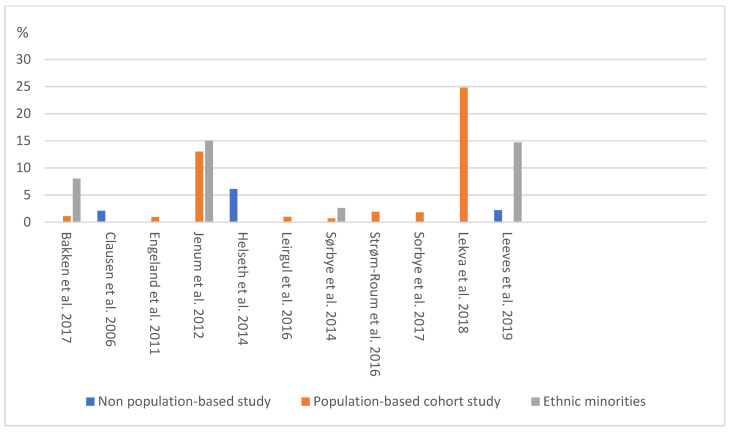
The prevalence of GDM in various population- and non-population-based studies.

In some selected studies, surveys were repeated over time or different surveys were conducted at different time points, allowing the estimation of changes in the prevalence of GDM over time. Although no published trend study was retrieved, the reported prevalence of GDM tended to be higher in recent years than before. In this respect, using the national criteria and database, Engeland et al. (2011) reported that the prevalence of GDM in Norway rose uniformly across different population groups between 2004 to 2008 from 0.8% to 1.2%, respectively [30]. Furthermore, Leeves, et al. (2019) reported that it increased from 1% in 2004 to 4% in 2015 [36]. A summary of the prevalence of GDM in various population- and non-population -based studies using the national criteria has been presented in Figure 2.

### 3.4. Quality Assessment and Risk of Bias Results

Details of the quality assessment of studies included are presented in the supplementary tables and figures. Accordingly, 90% of the studies were classified as high quality [26,29,30,32,33,34,35,36,37,38], and 9% as moderate [31], and no study had low quality. Overall, most of the studies were judged as having low risk of bias for the evaluated domains.

Cohort studies had a low risk of bias for the assessment of exposure, presence of the outcome of interest at the start of the study, outcome assessment, assessment of the presence or absence of prognostic factors, and adequacy of follow up of cohorts; however, one third of them had a high risk of bias in controlling prognostic variables and 14% of them had a probable risk of bias in the selection of exposed and non-exposed cohorts (Table S1, Figure S1).

All cross-sectional studies had a low risk of bias in the assessment of exposure and development of outcome of interest, but approximately one third of them had a high risk of selection of cases and controls, and control of prognostic variables (Table S2, Figure S2).

## 4. Discussion

This review aimed to collect data from the literature systematically and synthesize knowledge about the prevalence of GDM in Norway. The findings of 11 studies were summarized in this review, suggesting that the prevalence of GDM in population registered-based studies in Norway was lower than 2%, using the WHO 1999-criteria. On the other hand, studies on high-risk populations such as those of non-European ethnicity reported a prevalence rate ranging from 8% to 15%. It should be noted that these studies included heterogenous populations and mostly reported the prevalence of GDM in healthy mothers with singleton pregnancies. Our findings support the perspective that the prevalence of GDM have had an increasing trend in the recent decades. However, drawing a robust conclusion about the prevalence of GDM and its trend in Norway needs further population-based research.

GDM is one of the most important endocrinopathies during pregnancy [1]. The label of GDM brings with itself an intervention package consisting of glucose monitoring; extra clinic visits; more obstetric monitoring with greater likelihood of intervention; a label of high risk for developing metabolic disturbances and diabetes in the mother [39]. Therefore, it is of great public health significance to understand the feature of GDM with a national scope in each country.

Despite ethnic differences and variations in diagnostic criteria for GDM between countries, an increase in the prevalence of GDM and GDM-related risk factors has been observed in different healthcare settings. Therefore, it leaves no doubt that GDM has become a public health concern for many societies including developed western countries with a lower prevalence of GDM [40,41,42] with negative consequences for substantial short- and long-term adverse health outcomes for the mother and offspring [43,44]. Nevertheless, Nordic countries including Norway are known for having a relatively low prevalence of GDM on the global scale, which can be attributed to ethnicity, lower prevalence type 2 diabetes and obesity, and also a higher socioeconomic level compared to other nations across the globe [19,20].

According to the national report in Norway, the prevalence of GDM was 3–8% [24]. This estimation is higher than the estimation reported in other studies based on this database [30,32,33,34], but it is lower than the report of the recent published population-based non-registry study [26]. It can be assumed that the high percentage of disagreement between studies evaluating the prevalence of GDM can be explained by their methodological heterogeneities in terms of the study design, participants’ inclusion and exclusion criteria and parity, which explain the conflicting findings in our review. Moreover, the exact year of the conducted research may constitute an additional factor influencing the heterogeneity of the measured prevalence.

Our review showed an increasing trend of the prevalence of GDM during recent years in Norway. Similarly, other Nordic countries such as Denmark and Sweden [19,20] have reported the growing prevalence of GDM. However, increasing maternal age, along with the increasing rate of other risk factors such as diabetes, obesity and the growing number of women born outside of Scandinavia can be the underlying causes of the increasing rate of GDM [26,36,45,46].

According to our review, the prevalence of GDM among non-European pregnant women, particularly from the Middle East, south Asia and India was higher than that of the Western European population [47,48]. Norway is one of the European countries with in-migration from other ethnicities with a higher risk of GDM during recent years, which may potentially have affected the total prevalence of GDM in the general population. However, one of the most important factors influencing the prevalence of GDM is diagnostic criteria [2]. The Norwegian national GDM diagnostic criteria has been revised in 2017, which has recommended a lower glucose threshold for OGTT-75 g glucose. Up until now, only one study has assessed the prevalence of GDM based on the new criteria and has reported that 9.2% of pregnancies among low-risk women of Scandinavian heritage are affected by GDM [35].

The strength of this review lies in its concentration on scientific peer-reviewed published studies from Norway in both English and Norwegian language that have estimated GDM based on the recruitment of the large number of pregnant women. Also, the authors of this review sought to use a broad search strategy based on their previous experiences with the research topic and the process of systematic review as well as through the application of various general and specialized key words to identify all potentially eligible studies for inclusion. Furthermore, time and date restrictions were avoided to prevent selection bias. In addition, most of the included studies had high quality with low risk of bias, which led to a reliable conclusion of the selected studies’ findings. However, the available data were too diverse to yield a very concrete summary estimate of the prevalence of GDM. This was mainly due to the heterogeneity between inclusion criteria of the studies’ population that limited the comparability across the selected studies and hindered performing a meta-analysis. Moreover, since the new criteria for diagnosing GDM were established in 2017, there has not been a sufficient number of studies to assess the prevalence of GDM based on the new criteria.

## 5. Conclusions

Several aspects remain to be elucidated in order to completely clarify the prevalence of GDM and its trend in Norway. The overall prevalence of GDM in the low-risk population of Norway was reported low, but the evidence from the available literature supported the perspective that the prevalence of GDM has had an increasing trend in recent decades. Large-scale prospective cohort studies using the national registries data and also using new criteria are warranted to provide more robust evidence regarding the prevalence of GDM and its trend over the years, and assess the contribution of potential confounders including maternal age, BMI, and ethnicity. Our review findings can be used by policy makers in Norway and in other countries with a similar trend of GDM for devising appropriate health care strategies for improving women’s reproductive health.

## Figures and Tables

**Table 1:** The characteristics of the selected studies for data analysis and knowledge synthesis.

Authors, Year	Research Design	Eligibility Criteria of the Population	Data Resource	Research Year	GDM Diagnostic Criteria	Sample Size, *n*	GDM Prevalence *n* (%)
Bakken et al., (2017) [38]	Population-based study	Inclusion: First registered birth for women of Pakistani and Norwegian origin who delivered at Baerum Hospital Exclusion: Stillbirths cases, women of other country origin, type 1 diabetes, preterm labor before week 35, pregnancies with more than two fetuses, or fetuses with known health issues	Baerum Hospital and Medical Birth Registry of Norway and Statistics Norway	2006–2013	National Criteria: FBS ≥7.0 mmol/L and/or OGTT BS-2h ≥7.8 mmol/L	1. Norwegian origin: 8237 2. Pakistan origin: 287	1. 1. Norwegian origin: 1.1 2. Pakistan origin: 8.01
Clausen et al., (2006) [29]	Non population-based study	Inclusion: Norwegian-speaking women, living in Oslo city that had childbirth at Aker Hospital Exclusion: Type 1 diabetes, multiple pregnancies, abortion	Aker Hospital	1995–1997	National Criteria: OGTT BS-2h > 7.8 mmol/L	3677	78 (2.1)
Engeland et al., (2011) [30]	Population-based cohort study	Inclusion: First pregnancy, lasting more than 22 weeks Exclusion: Pregestational diabetes and hypertension, polycystic ovary syndrome	Medical Birth Registry of Norway and Norwegian Prescription Database	2004–2008	National Criteria: WHO-1999: FBS ≥7.0 mmol/L and/or OGTT BS-2h ≥7.8 mmol/L	1. 2004: 55,131 2. 2005: 52,529 3. 2006: 45,737 4: 2007: 38,039 6. 2008: 35,396 Total: 226,832	1. 2004: 472 (0.85) 2. 2005: 433 (0.82) 3. 2006: 478 (1.04) 4: 2007: 388 (1.02) 6. 2008: 427 (1.20) Total: 2198 (0.96)
Jenum, et al., (2012) [26]	Population-based study	Inclusion: Living in the districts, planned to give birth in the study hospitals, <20 weeks, ability to communicate and give written consent Exclusion: Pregestational diabetes or other diseases, need for intensive hospital follow-up during pregnancy	Three Public Child Health Clinics in Groruddalen in Oslo	2008–2010	1. National Criteria: WHO-1999: FBS ≥7.0 mmol/L and/or OGTT BS-2h ≥7.8 mmol/L: 2. modified IADPSG: FBS ≥5.1 mmol/L and/or OGTT BS-2h ≥8.5 mmol/L;	Total: 759 Western Europeans: 313 Ethnic minorities: 446	1. Total: 99 (13) 1. Western Europeans: 34 (11) 1. Ethnic minorities 67 (15) 2. Total: 239 (31.5) 2. Western Europeans: 75 (24) 2. Ethnic minorities 165 (37)
Helseth, et al., (2014) [31]	Non population-based study	Inclusion: Age ≥18 years, singleton live fetus Exclusion: High-risk pregnancies, diseases that interfered with participation in the study	St. Olavs Hospital, Trondheim University Hospital; Stavanger University Hospital	2007–2009	1. National Criteria: WHO-1999: FBS ≥7.0 mmol/L and/or OGTT BS-2h ≥7.8 mmol/L; 2. Simplified IADPSG: FBS ≥5.1 mmol/L and/or OGTT BS-2h ≥8.5 mmol/L	687	1. 42 (6.1) 2. 51 (7.4)
Leirgul et al., (2016) [32]	Population-based study	Inclusion: Registered birth Exclusion: Antidiabetic medication during pregnancy without a registered diabetes diagnosis, fetal chromosomal aberrations and relevant genetic disorders, multiple births	Medical Birth Registry of Norway	1994–2009	National Criteria: WHO-1999: FBS ≥7.0 mmol/L and/or OGTT BS-2h ≥7.8 mmol/L	914,427	9726 (1)
Sørbye et al., (2014) [37]	Population-based study	Inclusion: Singletons born Exclusion: Stillbirths <28 weeks, births that occurred before registered immigration, missing information on gestational length and cases with improbable birthweights based on gestational length and sex	Medical Birth Registry of Norway	1990–2009	National Criteria: FBS ≥7.0 mmol/L and/or OGTT BS-2h ≥7.8 mmol/L	1.Norway: 868,832 2. immigrant: 40,709	1. 0.7 2. 2.6
Strøm-Roum et al., (2016) [33]	Population-based study	Inclusion: Singleton pregnancies during the study period Exclusion: Missing information on maternal weight and height, offspring birthweight between 250–6500 g, recorded placental weight between 25–2500 g, pregnancy on weeks 22–45	Medical Birth Registry of Norway	2009–2012	National Criteria: WHO-1999: OGTT BS-2h ≥7.8–11 mmol/L	105,458	2078 (1.9)
Sorbye et al., (2017) [34]	Population-based study	Inclusion: Mothers with 1st and 2nd child Exclusion: Prepregnant BMI <15, interpregnancy weight change ±30	Medical Birth Registry of Norway	2006–2014	National Criteria: WHO-1999: OGTT BS-2h ≥7.8–11 mmol/L	24,198	439 (1.8)
Lekva, et al., (2018) [35]	Population-based study	Inclusion: Low-risk women of Scandinavian heritage Exclusion: Multiple pregnancies, known pre-gestational diabetes and any severe chronic diseases (lung, cardiac, gastrointestinal or renal).	Oslo University Hospital	2002–2008	1. WHO 2013: FBS ≥5.1 mmol/L, OGTT BS-1h ≥10 mmol/L, OGTT BS-2h ≥8.5 mmol/L; 2. 2017-Revised National criteria: FBS ≥5.3 mmol/L, OGTT BS-2h ≥9 mmol/L	1. 985; 2. 987	1. 244 (24.8) 2. 91 (9.2)
Leeves, et al., (2019) [36]	Population-based study	Inclusion: Women gave birth at study hospitals Exclusion: Multiple pregnancy	Hospitals in Nordland and Troms counties	2004–2015	National Criteria: FBS ≥7.0 mmol/L and/or OGTT BS-2h ≥7.8 mmol/L	1. 2004–2006: 7227; 2. 2007–2009: 9457; 3. 2010–2012:9318; 4. 2013–2015: 8913; 5. Total: 34,915; 6. non-European ethnicity: 755	1. 2004–2006: 72 (1) 2. 2007–2009: NM 3. 2010–2012: NM 4. 2013–2015: 356 (4) 5. Total: 782 (2.2) 6. non-European ethnicity: 111 (14.7)

GDM: gestational diabetes; FBS: fasting blood sugar; BS-2h: blood sugar 2 h; NM: Not Mentioned; WHO: World Health Organization; OGTT: oral glucose tolerance test; IADPSG: International Association of Diabetes and Pregnancy Study Group.

## Data Availability

The datasets used in this study are derived from publicly available data. The datasets analyzed are available from the corresponding author on reasonable request.

## Supplementary Materials

The following are available online at https://www.mdpi.com/1660-4601/18/4/1423/s1, Table S1. Quality assessment of the selected studies using the Newcastle–Ottawa Quality Assessment Scale for cohort studies, Table S2. Quality assessment of the selected studies using the Newcastle–Ottawa Quality Assessment Scale for cross-sectional studies, Figure S1. Risk of bias in cohort studies, Figure S2. Risk of bias in cross-sectional studies.

## References

[1] Saravanan P., Diabetes in Pregnancy Working Group, Maternal Medicine Clinical Study Group, Royal College of Obstetricians (2020). Gestational diabetes: Opportunities for improving maternal and child health. Lancet Diabetes Endocrinol..

[2] American Diabetes Association (2020). 2. Classification and diagnosis of diabetes: Standards of medical care in diabetes—2020. Diabetes Care.

[3] Behboudi-Gandevani S., Amiri M., Yarandi R.B., Tehrani F.R. (2019). The impact of diagnostic criteria for gestational diabetes on its prevalence: A systematic review and meta-analysis. Diabetol. Metab. Syndr..

[4] Lee K.W., Ching S.M., Ramachandran V., Yee A., Hoo F.K., Chia Y., Sulaiman W.A.W., Suppiah S., Mohamed M.H., Veettil S.K. (2018). Prevalence and risk factors of gestational diabetes mellitus in Asia: A systematic review and meta-analysis. BMC Pregnancy Childbirth.

[5] Muche A.A., Olayemi O.O., Kebede Y. (2019). Prevalence and determinants of gestational diabetes mellitus in Africa based on the updated international diagnostic criteria: A systematic review and meta-analysis. Arch. Public Health.

[6] Eades C.E., Cameron D.M., Evans J.M. (2017). Prevalence of gestational diabetes mellitus in Europe: A meta-analysis. Diabetes Res. Clin. Pr..

[7] Plows J.F., Stanley J.L., Baker P., Reynolds C.M., Vickers M.H. (2018). The Pathophysiology of Gestational Diabetes Mellitus. Int. J. Mol. Sci..

[8] Catalano P.M. (2014). Trying to understand gestational diabetes. Diabet. Med..

[9] Barbour L.A., McCurdy C.E., Hernandez T.L., Kirwan J.P., Catalano P.M., Friedman J.E. (2007). Cellular Mechanisms for Insulin Resistance in Normal Pregnancy and Gestational Diabetes. Diabetes Care.

[10] Sonagra A.D., Biradar S.M., Dattatreya K., DS J.M. (2014). Normal Pregnancy—A State of Insulin Resistance. J. Clin. Diagn. Res..

[11] Domanski G., Lange A.E., Ittermann T., Allenberg H., Spoo R.A., Zygmunt M., Heckmann M. (2018). Evaluation of neonatal and maternal morbidity in mothers with gestational diabetes: A population-based study. BMC Pregnancy Childbirth.

[12] Vounzoulaki E., Khunti K., Abner S.C., Tan B.K., Davies M.J., Gillies C.L. (2020). Progression to type 2 diabetes in women with a known history of gestational diabetes: Systematic review and meta-analysis. BMJ.

[13] Kramer C.K., Campbell S., Retnakaran R. (2019). Gestational diabetes and the risk of cardiovascular disease in women: A systematic review and meta-analysis. Diabetologia.

[14] Xu Y., Shen S., Sun L., Yang H., Jin B., Cao X. (2014). Metabolic Syndrome Risk after Gestational Diabetes: A Systematic Review and Meta-Analysis. PLoS ONE.

[15] Behboudi-Gandevani S., Tehrani F.R., Rahmati M., Amiri M., Azizi F. (2019). Trend of various adiposity indices in women with and without history of gestational diabetes: A population-based cohort study. BMC Endocr. Disord..

[16] McMahon L.E., O’Malley E.G., Reynolds C.M.E., Turner M.J. (2020). The impact of revised diagnostic criteria on hospital trends in gestational diabetes mellitus rates in a high income country. BMC Health Serv. Res..

[17] Ferrara A. (2007). Increasing Prevalence of Gestational Diabetes Mellitus: A public health perspective. Diabetes Care.

[18] Brown F.M., Wyckoff J. (2017). Application of One-Step IADPSG Versus Two-Step Diagnostic Criteria for Gestational Diabetes in the Real World: Impact on Health Services, Clinical Care, and Outcomes. Curr. Diabetes Rep..

[19] Fadl H.E., Simmons D. (2016). Trends in diabetes in pregnancy in Sweden 1998–2012. BMJ Open Diabetes Res. Care.

[20] Jeppesen C., Maindal H.T., Kristensen J.K., Ovesen P.G., Witte D.R. (2017). National study of the prevalence of gestational diabetes mellitus among Danish women from 2004 to 2012. Scand. J. Public Health.

[21] Statistics Norway (2021). Mean Age of Parent at First Child’s Birth 1961–2019. https://www.ssb.no/en/statbank/table/07872.

[22] Norwegian Institute for Public Health (2017). Diabetes-in-Norway. https://www.fhi.no/en/op/hin/health-disease/diabetes-in-norway---public-health-/.

[23] Helsedirektoratet (2020). Diabetes: Retningslinjer om Diabetes og Svangerskapsdiabetes. Opplæringsmateriell til helsepersonell. https://www.helsedirektoratet.no/retningslinjer/svangerskapsdiabetes.

[24] The Norwegian Institute of Public Health (2019). The Effectiveness of Screening all Pregnant Women Versus Pregnant Women with Risk Factors for Gestational Diabetes. https://www.fhi.no/en/publ/2019/effekten-av-a-screene-alle-gravide-sammenlignet-med-a-screene-gravide-med-r/.

[25] Borgen I., Garnweidner-Holme L., Jacobsen A.F., Fayyad S., Småstuen M.C., Lukasse M. (2019). Knowledge of gestational diabetes mellitus at first consultation in a multi-ethnic pregnant population in the Oslo region, Norway—A cross-sectional study. Ethn. Health.

[26] Jenum A.K., Mørkrid K., Sletner L., Vangen S., Torper J.L., Nakstad B., Voldner N., Rognerud-Jensen O.H., Berntsen S., Mosdøl A. (2012). Impact of ethnicity on gestational diabetes identified with the WHO and the modified International Association of Diabetes and Pregnancy Study Groups criteria: A population-based cohort study. Eur. J. Endocrinol..

[27] Moher D., Liberati A., Tetzlaff J., Altman D.G., PRISMA Group (2009). Preferred reporting items for systematic reviews and meta-analyses: The PRISMA statement. BMJ.

[28] Wells G.A., Shea B., O’Connell D., Peterson J., Welch V., Losos M., Tugwell P. The Newcastle-Ottawa Scale (NOS) for Assessing the Quality of Nonrandomized Studies in Meta-Analyses.

[29] Clausen T., Øyen N., Henriksen T. (2006). Pregnancy complications by overweight and residential area. A prospective study of an urban Norwegian cohort. Acta Obstet. Gynecol. Scand..

[30] Engeland A., Bjørge T., Daltveit A.K., Skurtveit S., Vangen S., Vollset S.E., Furu K. (2011). Risk of diabetes after gestational diabetes and preeclampsia. A registry-based study of 230,000 women in Norway. Eur. J. Epidemiol..

[31] Helseth R., Salvesen Ø., Stafne S.N., Mørkved S., Salvesen K.Å., Carlsen S.M. (2014). Gestational diabetes mellitus among Nordic Caucasian women: Prevalence and risk factors according to WHO and simplified IADPSG criteria. Scand. J. Clin. Lab. Investig..

[32] Leirgul E., Brodwall K., Greve G., Vollset S.E., Holmstrøm H., Tell G.S., Øyen N. (2016). Maternal Diabetes, Birth Weight, and Neonatal Risk of Congenital Heart Defects in Norway, 1994–2009. Obstet. Gynecol..

[33] Strøm-Roum E.M., Tanbo T., Eskild A. (2016). The associations of maternal body mass index with birthweight and placental weight. Does maternal diabetes matter? A population study of 106 191 pregnancies. Acta Obstet. Gynecol. Scand..

[34] Sørbye L.M., Skjaerven R., Klungsøyr K., Morken N.-H. (2017). Gestational diabetes mellitus and interpregnancy weight change: A population-based cohort study. PLoS Med..

[35] Lekva T., Godang K., Michelsen A.E., Qvigstad E., Normann K.R., Norwitz E.R., Aukrust P., Henriksen T., Bollerslev J., Roland M.C.P. (2018). Prediction of Gestational Diabetes Mellitus and Pre-diabetes 5 Years Postpartum using 75 g Oral Glucose Tolerance Test at 14-16 Weeks’ Gestation. Sci. Rep..

[36] Leeves L.T., Andreasen C., Marrable S., Glasø M.U., Rostad M.-K., Olsen I.P., Bjørnerem Å. (2019). Prevalens av diabetes blant gravide og svangerskapsutfall i Nordland og Troms 2004–15. Tidsskr. Den Nor. Legeforening.

[37] Sørbye I.K., Daltveit A.K., Sundby J., Vangen S. (2014). Preterm subtypes by immigrants’ length of residence in Norway: A population-based study. BMC Pregnancy Childbirth.

[38] Bakken K.S., Skjeldal O.H., Stray-Pedersen B. (2015). Obstetric Outcomes of First- and Second-Generation Pakistani Immigrants: A Comparison Study at a Low-Risk Maternity Ward in Norway. J. Immigr. Minor. Health.

[39] Poolsup N., Suksomboon N., Amin M. (2014). Effect of Treatment of Gestational Diabetes Mellitus: A Systematic Review and Meta-Analysis. PLoS ONE.

[40] Lavery J.A., Friedman A.M., Keyes K.M., Wright J.D., Ananth C.V. (2017). Gestational diabetes in the United States: Temporal changes in prevalence rates between 1979 and 2010. BJOG: Int. J. Obstet. Gynaecol..

[41] Zhu Y., Zhang C.-L. (2016). Prevalence of Gestational Diabetes and Risk of Progression to Type 2 Diabetes: A Global Perspective. Curr. Diabetes Rep..

[42] Buckley B.S., Harreiter J., Damm P., Corcoy R., Chico A., Simmons D., Vellinga A., Dunne F., on behalf of the DALI Core Investigator group (2012). Gestational diabetes mellitus in Europe: Prevalence, current screening practice and barriers to screening. A review. Diabet. Med..

[43] Nijs H., Benhalima K. (2020). Gestational Diabetes Mellitus and the Long-Term Risk for Glucose Intolerance and Overweight in the Offspring: A Narrative Review. J. Clin. Med..

[44] Billionnet C., Mitanchez D., Weill A., Nizard J., Alla F., Hartemann A., Jacqueminet S. (2017). Gestational diabetes and adverse perinatal outcomes from 716,152 births in France in 2012. Diabetologia.

[45] Miao M., Dai M., Zhang Y., Sun F., Guo X., Sun G. (2017). Influence of maternal overweight, obesity and gestational weight gain on the perinatal outcomes in women with gestational diabetes mellitus. Sci. Rep..

[46] Kuo C.-H., Chen S.-C., Fang C.-T., Nien F.-J., Wu E.-T., Lin S.-Y., Chuang L.-M., Lee C.-N., Li H. (2017). Screening gestational diabetes mellitus: The role of maternal age. PLoS ONE.

[47] Hedderson M.M., Darbinian J.A., Ferrara A. (2010). Disparities in the risk of gestational diabetes by race-ethnicity and country of birth. Paediatr. Périnat. Epidemiol..

[48] Pu J., Zhao B., Wang E.J., Nimbal V., Osmundson S., Kunz L., Popat R.A., Chung S., Palaniappan L.P. (2015). Racial/Ethnic Differences in Gestational Diabetes Prevalence and Contribution of Common Risk Factors. Paediatr. Périnat. Epidemiol..

