# Supplementary material for: A Systematic Review of the Prevalence of Gestational Diabetes in Norway
Source: Int J Environ Res Public Health. 2021 Feb 3;18(4):1423. doi: 10.3390/ijerph18041423 (PMC7913746; doi:10.3390/ijerph18041423)
Supplement: Supplementary file 1 [file ijerph-18-01423-s001.pdf]

Table S1. Quality assessment of the selected studies using the Newcastle–Ottawa Quality Assessment Scale for cohort studies.

Author, Year	Selection (Maximum Four *)				Comparability (Maximum Two *)	Outcome (Maximum Three *)			Total Scores
	Representativeness of The Exposed Cohort *	Selection of The Non-Exposed Cohort *	Ascertainment of Exposure *	No Outcome of Interest at Start of The Study *	Controlled for confounders: A: Age and/or BMI * B: others *	Assessment of outcome (maximum one *) A: Doctor's Diagnosis OR Objective Measurements * B: Parent/Self-Reported Doctor's Diagnosis OR Use of Medication *	Follow-Up Long Enough for Outcomes *	Adequacy of Follow Up of Cohorts *	
Clausen et al. (2006)			*	*	**	*	*	*	7 *
Engeland et al. (2011)	*	*	*	*	*	*	*	*	8 *
Jenum et al. (2012)	*	*	*	*	**	*	*	*	9 *
Leirgul et al. (2016)	*	*	*	*	**	*	*	*	9 *
Sorbye et al. (2017	*	*	*	*	**	*	*	*	9 *
Lekva et al. (2018)	*	*	*	*	*	*	*	*	8 *
Leeves et al. (2019)	*	*	*	*	**	*	*	*	9 *

BMI: body mass index.

Table S2. Quality assessment of the selected studies using the Newcastle–Ottawa Quality Assessment Scale for cross-sectional studies.

Author, Year	Selection (Maximum Four *)				Comparability (Maximum Two *)	Outcome (Maximum Three *)		Total Scores
	Representativeness of The Samples *	Sample Size *	Non-Responders *	Ascertainment of The Exposure (Risk Factor) *	Controlled for confounders: A: Age and/or BMI * B: others *	Assessment of The Outcome (maximum two *) a) Independent Blind Assessment ** b) Record Linkage ** c) Self Report *	Statistical Test *	
Bakken et al. (2017)	*	*		*	**	**	*	8 *
Helseth et al. (2014)				*		**	*	4 *
Sørbye et al. (2014)	*	*		*	**	**	*	8 *
Strøm-Roum et al. (2016)	*			*	**	**	*	7 *

BMI: body mass index.

Author Year	Bias in The Selection of Exposed and Non-Exposed Cohorts	Bias in The Assessment of Exposure	Bias in The Presence of Outcome of Interest at Start of The Study	Bias in The Control of Prognostic Variables (with Matching or Adjusting)	Bias in The Assessment of The Presence or Absence of Prognostic Factors	Bias in The Assessment of Outcome	Bias in Adequacy in Follow Up of Cohorts
Clausen et al. (2006)	Definitely yes (high risk of bias)	Definitely No (low risk of bias)	Definitely No (low risk of bias)	Definitely No (low risk of bias)	Definitely No (low risk of bias)	Definitely No (low risk of bias)	Definitely No (low risk of bias)
Engeland et al. (2011)	Definitely No (low risk of bias)	Definitely No (low risk of bias)	Definitely No (low risk of bias)	Probably no	Definitely No (low risk of bias)	Definitely No (low risk of bias)	Definitely No (low risk of bias)
Jenum, et al. (2012)	Definitely No (low risk of bias)	Definitely No (low risk of bias)	Definitely No (low risk of bias)	Definitely No (low risk of bias)	Definitely No (low risk of bias)	Definitely No (low risk of bias)	Definitely No (low risk of bias)
Leirgul et al. (2016)	Definitely No (low risk of bias)	Definitely No (low risk of bias)	Definitely No (low risk of bias)	Definitely No (low risk of bias)	Definitely No (low risk of bias)	Definitely No (low risk of bias)	Definitely No (low risk of bias)
Sorbye et al. (2017)	Definitely No (low risk of bias)	Definitely No (low risk of bias)	Definitely No (low risk of bias)	Definitely No (low risk of bias)	Definitely No (low risk of bias)	Definitely No (low risk of bias)	Definitely No (low risk of bias)
Lekva et al. (2018)	Definitely No (low risk of bias)	Definitely No (low risk of bias)	Definitely No (low risk of bias)	Probably no	Definitely No (low risk of bias)	Definitely No (low risk of bias)	Definitely No (low risk of bias)
Leeves et al. (2019)	Definitely No (low risk of bias)	Definitely No (low risk of bias)	Definitely No (low risk of bias)	Definitely No (low risk of bias)	Definitely No (low risk of bias)	Definitely No (low risk of bias)	Definitely No (low risk of bias)

Definitely No (low risk of bias)   Probably no

Definitely yes (high risk of bias)   Probably Yes

(A)

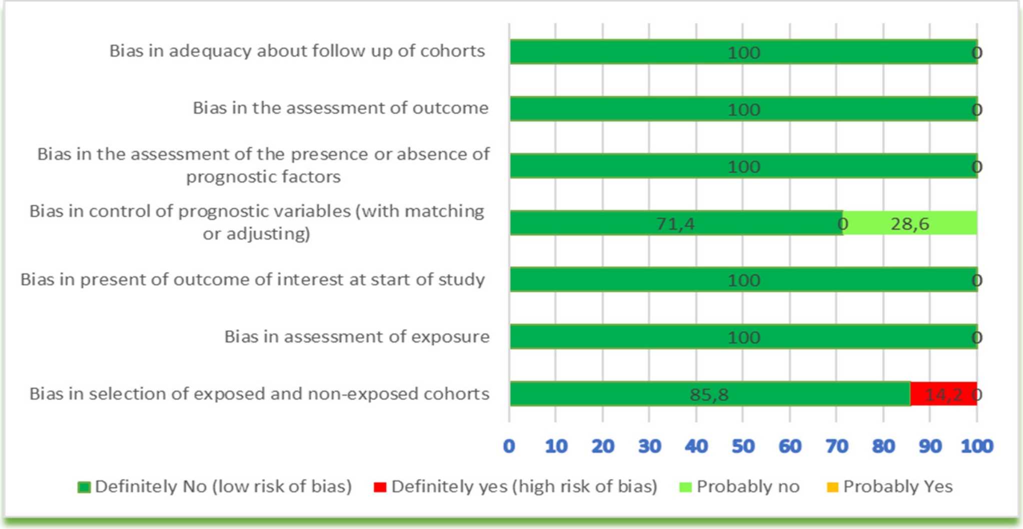

(B)

Figure S1. Risk of bias in cohort studies.

Author, Year	Bias in The Assessment of Exposure (Risk Factor)	Bias in The Development of Outcome of Interest in Case and Controls	Bias in The Selection of Cases	Bias in The Selection of Controls	Bias in The Control of Prognostic Variable (without Case and Control Matching or Adjustment in Statistical Methods)
Bakken et al. (2017)	🟢	🟢	🟢	🟢	🟢
Helseth et al. (2014)	🟢	🟢	🔴	🔴	🔴
Sørbye et al. (2014)	🟢	🟢	🟢	🟢	🟢
Strøm-Roum et al. (2016)	🟢	🟢	🟢	🟢	🟢

🟢 Definitely No (low risk of bias) — Probably no

🔴 Definitely yes (high risk of bias) — Probably Yes

(A)

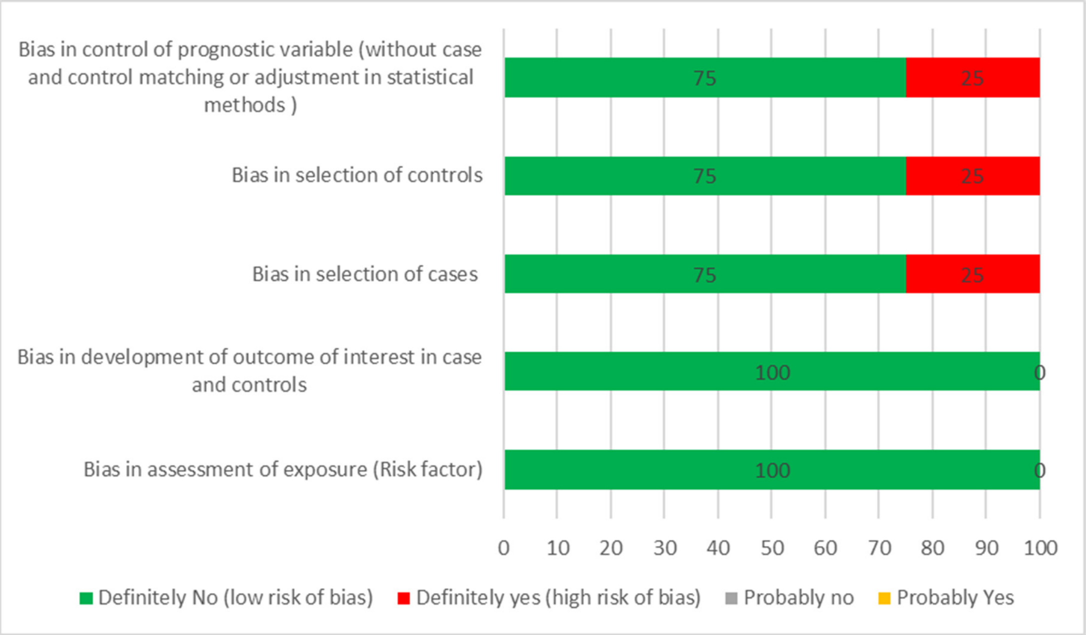

(B)

Figure S2. Risk of bias in cross-sectional studies.
